# Knowledge, attitudes, and practices among Chinese reproductive-age women toward uterine adenomyosis

**DOI:** 10.3389/fmed.2024.1361671

**Published:** 2024-04-08

**Authors:** Ruofei Ren, Hongye Li, Jie Zhang, Xuhua Li, Liqing Yuan, Dongxiao Li, Shuzhi Shan, Bin Shi, Jing Jiang

**Affiliations:** Department of Obstetrics and Gynecology, The Second Hospital of Hebei Medical University, Shijiazhuang, Hebei, China

**Keywords:** knowledge, attitudes, practices, cross-sectional study, uterine adenomyosis

## Abstract

**Objective:**

This study aimed to assess the knowledge, attitudes and practices (KAP) among Chinese reproductive-age women toward uterine adenomyosis.

**Methods:**

This web-based cross-sectional study was conducted between April 2023 and September 2023 at the Second Hospital of Hebei Medical University. A self-designed questionnaire was developed to collect demographic information of reproductive-age women, and assess their KAP toward uterine adenomyosis.

**Results:**

A total of 520 valid questionnaires were collected. Among the participants, 127 (24.42%) were diagnosed with uterine adenomyosis, and 120 (23.08%) were accompanied by uterine fibroids. The mean knowledge, attitudes and practices scores were 3.54 ± 3.72 (possible range:0–10), 20.96 ± 3.19 (possible range:5–25) and 24.01 ± 4.95 (possible range:7–35), respectively. The structural equation model demonstrated that knowledge had direct effects on attitudes and practices, as indicated by a path coefficient of 0.714 (*p* < 0.001) and 1.510 (*p* < 0.001), respectively. Moreover, attitudes had direct effects on practices, with a path coefficient of 0.226 (*p* = 0.001).

**Conclusion:**

The findings revealed that reproductive-age women have insufficient knowledge, negative attitudes, and poor practices toward the uterine adenomyosis. Comprehensive training programs are needed to improve reproductive-age women practices in this area.

## Introduction

Uterine adenomyosis is a significant gynecological condition characterized by the invasive growth of endometrial tissues into the uterine muscle, resembling malignant tumors in its implantation and recurrence patterns (1, 2). This condition impacts women’s health, frequently manifesting through symptoms like dysmenorrhea, chronic pelvic pain, and infertility (3). In China, the prevalence of adenomyosis among women varies widely, reported between 5% and 70%. The condition poses a significant burden not only on the affected women, often leading to chronic pain and reproductive issues, but also on the healthcare system, due to the costs associated with its management and treatment (4).

Adenomyosis is increasingly identified in women of reproductive age due to advancements in diagnostic imaging and clinical evidence of pelvic pain, abnormal uterine bleeding, and infertility (5). However, diagnosis remains difficult and unclear, making it a clinically neglected condition. This lack of clarity in diagnosis and the varying symptoms contribute to the gap in knowledge and awareness among Chinese women (6). Additionally, distinguishing adenomyosis from other gynecological malignancies is crucial for accurate diagnosis and treatment. For example, the differential diagnosis of uterine sarcomas, which may mimic adenomyosis but require different management strategies (7). A deficiency in the knowledge base regarding this condition may cause undue alarm among women concerning their health. Furthermore, a study focused on the prevention and early diagnosis of gynecological cancers suggests that patients should be encouraged to conduct self-examinations and to be vigilant for the emergence of symptoms. It further recommends that women should adopt healthy lifestyles and maintain balanced diets as part of their routine management (8). The studies mentioned above collectively underscore the importance of conducting precise surveys on the current state of knowledge and attitudes toward adenomyosis among women, as well as examining their practices in health behaviors. Such investigations are crucial for a deeper understanding of their current situation regarding disease management. However, there is a notable scarcity of comprehensive research in this area.

The concept of Knowledge, Attitudes, and Practices (KAP) research provides a framework for comprehensively investigating individuals’ awareness, beliefs, and behaviors related to a specific health condition (9). In the context of uterine adenomyosis, understanding the KAP of reproductive-age Chinese women becomes pivotal for devising effective public health strategies and enhancing patient-centered care.

## Materials and methods

### Study design and participants

This web-based cross-sectional survey was conducted between April 2023 and September 2023 at the Second Hospital of Hebei Medical University. This study was approved by the Ethics Committee of the Second Hospital of Hebei Medical University (Approval No. 2023-R010), and all participants provided written informed consent.

The inclusion criteria were as follows: (1) women of reproductive age, specifically aged between 15 and 49 years. (2) capability to comprehend and complete the relevant questionnaire surveys. Those who were currently pregnant or in the lactation period, as well as those with severe psychological or physical illnesses, including gynecological tumors, were excluded from the study.

### Questionnaire

The questionnaire was developed with guidance from the expert consensus on the uterine adenomyosis and pertinent literatures (10–12). A preliminary trial was conducted on a small scale (*n* = 26), resulting in a Cronbach’s alpha coefficient value of 0.872, indicating good internal reliability.

The final questionnaire was in Chinese and consisted of four dimensions: demographic information, knowledge, attitudes, and practices. The demographic section consisted of 20 items, while the knowledge, attitude, and practice dimensions included 12, 6, and 7 items, respectively. To ensure data quality, questions K3 and K9 were strategically designed as trap questions, presenting diametrically opposite meanings, and neither of these two questions was scored. Respondents who selected contradictory answers for both questions were identified as having a logical conflict and were thus excluded from the survey. The knowledge items were scored 1 point for a correct answer and 0 points for others, resulting in a possible score range of 0–10. For attitude assessment, a five-point Likert scale ranging from very positive (5 points) to very negative (1 point) was utilized, with questions A6 and 6.1 earmarked exclusively for descriptive analysis, producing a possible score range of 5 to 25. Similarly, the practice items were also scored on a five-point Likert scale, spanning from very consistent/always (5 points) to very inconsistent/never (1 point), resulting in a potential score range of 7 to 35.

Data collection was facilitated through an online questionnaire hosted on Sojump.^1^ The distribution of the online questionnaire was executed via diverse social media platforms, ensuring broad accessibility. Prior to engaging with the e-questionnaire, participants were required to affirm their willingness to participate by selecting the option “I agree to participate in this study.” To maintain participant anonymity, all data were collected anonymously. To prevent redundancy, IP restriction measures were implemented, restricting completion of the survey to a single occurrence from a specific IP address.

### Statistical analysis

STATA 17.0 (Stata Corporation, College Station, TX, United States) was used for statistical analysis. The continuous variables were expressed as mean ± SD, and the categorical variables was expressed as *n* (%). The continuous variables conformed to a normal distribution were tested by the *t*-test or ANOVA. This study used the scores distribution from 70% of the recovered valid questionnaires as the cutoff values for the three dimensions: Knowledge (with a threshold score of 6 points), Attitude (with a threshold score of 22 points), and Practice (with a threshold score of 26 points). Pearson correlation was used to analyze the correlation between knowledge, attitudes, and practices. The structural equation model (SEM) of knowledge, attitudes and practices among reproductive-age women toward uterine adenomyosis was constructed with AMOS 24.0 (IBM, NY, United States). The hypotheses as following: (1) knowledge had direct effects on attitude, (2) knowledge had direct effects on practice, and (3) attitude had direct effects on practice. The model fitting was evaluated with CMIN/DF (Chi-square fit statistics/degree of freedom), RMSEA (root mean square error of approximation), IFI (incremental fix index), TLI (Tucker-Lewis index) and CFI (comparative fix index). In this analysis, *p* < 0.05 was considered statistically significant.

## Results

This study initially garnered a total of 605 questionnaires. Subsequently, a portion of these questionnaires were disqualified due to either logical inconsistencies or incomplete submissions within the designated timeframe. Ultimately, this resulted in a total of 520 valid responses being retained for analysis. Among them, 327(62.88%) lived in urban areas, with mean age of 35.78 ± 8.33 years, 292 (56.15%) had BMI in the range of 18.5–23.9 kg/m^2^. Furthermore, 405 (77.88%) were married, with mean menstrual cycle of 30.37 ± 17.98 days, and mean menstrual period of 6.02 ± 1.44 days. 144 (27.69%) had no experience of childbirth, 242 (46.54%) had no experience of abortion, 281 (54.04%) had symptoms of dysmenorrhoea and 127 (24.42%) were diagnosed with uterine adenomyosis. The mean knowledge, attitude and practice scores were 3.54 ± 3.72 (possible range:0–10), 20.96 ± 3.19 (possible range:5–25) and 24.01 ± 4.95 (possible range:7–35), respectively. Higher knowledge scores were found in participants who lived in urban areas (*p* = 0.015), single (*p* < 0.001), had a monthly income of 10,000–20,000 (CNY) (*p* = 0.018), had no abortion (*p* = 0.040), experienced dysmenorrhea symptoms (*p* < 0.001), had worsening dysmenorrhea (*p* < 0.001), and had a pain score of 1–3 at the worst stage of dysmenorrhea (*p* < 0.001). Meanwhile, higher attitudes scores were also found among participants who lived in urban areas (*p* < 0.001). Higher practice scores were also found in those participants with a BMI of less than 18.5 kg/m^2^ (*p* = 0.030), experienced dysmenorrhea symptoms (*p* = 0.001), had worsening dysmenorrhea (*p* < 0.001), and had a pain score of 4–6 at the worst stage of dysmenorrhea (*p* < 0.001). Significantly, individuals holding a master’s degree or above (*p* < 0.001, *p* < 0.001, *p* = 0.019, separately) as well as those diagnosed uterine adenomyosis (*p* < 0.001, *p* = 0.031, *p* < 0.001, separately) demonstrated elevated levels of knowledge, attitude, and practice scores (Table 1).

**Table 1:** Demographic characteristics.

	*N* (%)	Knowledge		Attitude		Practice	
		Mean ± SD	*P*	Mean ± SD	*P*	Mean ± SD	*P*
Total	520	3.54 ± 3.72		20.96 ± 3.19		24.01 ± 4.95	
Age (years)	35.78 ± 8.33						
BMI (kg/m^2^)			0.749		0.188		0.030
<18.5	32 (6.15)	3.16 ± 3.87		20.16 ± 5.04		25.87 ± 4.94	
18.5–23.9	292 (56.15)	3.63 ± 3.82		21.14 ± 3.24		24.16 ± 4.84	
≥24	196 (37.69)	3.46 ± 3.55		20.82 ± 2.68		23.48 ± 5.06	
Residence			0.015		<0.001		0.147
Rural	193 (37.12)	3.02 ± 3.63		20.33 ± 3.30		23.60 ± 5.28	
Urban	327 (62.88)	3.84 ± 3.74		21.33 ± 3.06		24.25 ± 4.74	
Marital status			<0.001		0.191		0.618
Single	107 (20.58)	4.93 ± 4.10		21.45 ± 4.45		24.39 ± 5.71	
Married	405 (77.88)	3.18 ± 3.54		20.82 ± 2.77		23.90 ± 4.76	
Divorced/Other	8 (1.54)	3.13 ± 3.40		21.25 ± 2.31		24.63 ± 3.78	
Education level			<0.001		<0.001		0.019
Primary school and below	114 (21.92)	1.95 ± 2.96		20.08 ± 2.32		23.14 ± 4.11	
High school and vocational school	87 (16.73)	3.09 ± 3.31		20.34 ± 2.28		24.00 ± 4.43	
Junior college and bachelor’s degree	254 (48.85)	3.59 ± 3.69		21.12 ± 3.64		24.01 ± 5.35	
Master’s degree and above	65 (12.50)	6.69 ± 3.65		22.69 ± 2.93		25.57 ± 5.08	
Average monthly income (CNY)			0.018		0.079		0.721
<2,000	46 (8.85)	4.54 ± 3.50		21.63 ± 2.72		24.26 ± 5.78	
2,000–5,000	246 (47.31)	3.08 ± 3.64		20.69 ± 2.92		23.87 ± 4.60	
5,000–10,000	161 (30.96)	3.57 ± 3.77		20.88 ± 3.37		23.89 ± 5.08	
10,000–20,000	57 (10.96)	4.61 ± 3.89		21.53 ± 4.00		24.84 ± 5.47	
>20,000	10 (1.92)	3.50 ± 3.24		22.60 ± 2.37		23.60 ± 4.79	
Occupation			0.224		0.260		0.506
Regular employee/part-time/self-employed	331 (63.65)	3.39 ± 3.65		21.08 ± 3.17		24.12 ± 4.88	
Unemployed/retired/other	189 (36.35)	3.80 ± 3.83		20.75 ± 3.21		23.82 ± 5.09	
Medical insurance			0.500		0.143		0.081
No medical insurance	32 (6.15)	4.22 ± 3.60		20.63 ± 4.16		23.50 ± 4.92	
Social insurance only	314 (60.38)	3.43 ± 3.71		20.78 ± 3.08		23.68 ± 4.92	
Social insurance and other commercial insurance	174 (33.46)	3.60 ± 3.76		21.34 ± 3.21		24.70 ± 4.99	
Menstrual age (years)	13.43 ± 1.37						
Menstrual cycle (days)	30.37 ± 17.98						
Menstrual period (days)	6.02 ± 1.44						
Menstrual flow			0.116		0.706		0.058
Scanty, spotting	42 (8.08)	3.81 ± 3.83		20.83 ± 3.35		23.64 ± 4.84	
Moderate, normal amount	387 (74.42)	3.35 ± 3.70		20.91 ± 3.33		23.79 ± 5.00	
Heavy, affecting quality of life	91 (17.50)	4.22 ± 3.71		21.21 ± 2.40		25.13 ± 4.69	
**Reproductive history (multiple choices)**							
None	144 (27.69)	4.38 ± 4.02		21.26 ± 4.27		24.13 ± 5.71	
Vaginal delivery	218 (41.92)	3.13 ± 3.45		20.87 ± 2.44		24.08 ± 4.20	
Cesarean section	179 (34.42)	3.46 ± 3.71		20.84 ± 2.89		24.02 ± 5.12	
Count of miscarriage incidents (encompassing both surgical interventions and medication-induced terminations)			0.040		0.124		0.809
No history of miscarriage	242 (46.54)	3.96 ± 3.98		21.26 ± 3.67		24.11 ± 5.45	
1 time	139 (26.73)	3.31 ± 3.47		20.82 ± 2.59		23.96 ± 4.67	
2 times	90 (17.31)	2.58 ± 3.30		20.39 ± 2.48		23.56 ± 4.17	
3 times	34 (6.54)	3.88 ± 3.40		20.50 ± 3.43		24.74 ± 4.83	
4 times or more	15 (2.88)	3.80 ± 3.91		21.87 ± 2.47		23.93 ± 3.81	
Experienced dysmenorrhea symptoms			<0.001		0.839		0.001
Yes	281 (54.04)	4.08 ± 3.75		20.99 ± 3.32		24.69 ± 5.08	
No	239 (45.96)	2.90 ± 3.58		20.93 ± 3.03		23.21 ± 4.69	
^*^Dysmenorrhea worsen progressively			<0.001		0.769		<0.001
*Yes*	88 (16.92)	5.40 ± 3.50		21.18 ± 2.95		26.02 ± 4.38	
*No*	193 (37.12)	3.48 ± 3.72		20.90 ± 3.48		24.08 ± 5.27	
^*^Dysmenorrhea severity rating at its worst			<0.001		0.115		<0.001
*0 points, no pain*	176 (33.85)	3.31 ± 3.67		20.67 ± 3.24		24.07 ± 5.16	
*1–3 points, some pain but tolerable*	69 (13.27)	5.45 ± 3.73		21.26 ± 3.77		25.61 ± 5.18	
*4–6 points, noticeable pain, requires pain relief medication*	36 (6.92)	5.22 ± 3.24		22.00 ± 2.51		25.97 ± 4.01	
*7–10 points, severe pain, significantly affects sleep, requires pain relief medication*	0	-				-	
Diagnosed with uterine adenomyosis			<0.001		0.031		<0.001
Yes	127 (24.42)	5.80 ± 3.12		21.49 ± 2.15		26.46 ± 3.67	
No	393 (75.58)	2.81 ± 3.60		20.79 ± 3.44		23.22 ± 5.06	
Other gynecological conditions apart from uterine adenomyosis (multiple choices)							
Endometriosis, ovarian cysts	64 (12.31)	5.38 ± 3.47		22.00 ± 2.58		25.80 ± 4.17	
Uterine fibroids	120 (23.08)	3.43 ± 3.57		21.07 ± 2.40		24.33 ± 4.08	
Inflammatory conditions: e.g., vulvovaginitis, cervicitis, pelvic inflammatory disease, etc.	57 (10.96)	2.40 ± 3.35		21.07 ± 3.17		22.96 ± 3.64	
Endocrine disorders: menstrual irregularities, polycystic ovary syndrome, etc.	21 (4.04)	4.38 ± 3.49		21.24 ± 5.86		25.19 ± 6.12	
Malignant tumors: cervical cancer, endometrial cancer, ovarian cancer, etc.	1 (0.19)	5.00		25.00		29.00	
None of the above	288 (55.38)	3.44 ± 3.79		20.82 ± 3.27		23.74 ± 5.36	

^*^Participants were instructed to answer the section in italics only if they selected “Yes” in response to the question regarding the presence of dysmenorrhea.

The knowledge dimension revealed that the correctness rates of participants’ answers to all questions did not exceed 50%, the three knowledge items with the highest correctness rates were as follows: “Adenomyosis is not typically associated with the induction of psychological or physiological discomfort in women.” (K9) with 48.85%, “Adenomyosis can lead to both psychological and physiological distress in affected women.” (K3) with 48.27%, and “A healthy diet, regular exercise, and consistent follow-up can ameliorate the condition of patients with adenomyosis.” (K11) with 46.35%. The three items with the lowest correctness rates were: “Therapeutic interventions for adenomyosis may involve pharmacological treatments or surgical options. Post-remission, symptoms will never recur.” (K5) with 18.46%, “Adenomyosis does not possess malignant transformation potential.” (K12) with 27.12%, and “For patients experiencing symptomatic adenomyosis and pursuing definitive resolution, hysterectomy may be considered” (K7) with 28.27% (Table 2).

**Table 2:** Knowledge.

	Correctness *N* (%)
K1. Typical clinical manifestations of adenomyosis encompass menorrhagia and dysmenorrhea. (**correct**)	230 (44.23)
K2. Women of reproductive age are not the primary demographic impacted by adenomyosis. (**incorrect**)	153 (29.23)
K3. Adenomyosis can lead to both psychological and physiological distress in affected women. (**correct**)	251 (48.27)
K4. Diagnostic approaches for adenomyosis include ultrasonography and magnetic resonance imaging (MRI). (**correct**)	236 (45.38)
K5. Therapeutic interventions for adenomyosis may involve pharmacological treatments or surgical options. Post-remission, symptoms will never recur. (**incorrect**)	96 (18.46)
K6. Pharmacological management of adenomyosis often entails the use of analgesics, oral contraceptives, and progestogens. Certain Traditional Chinese Medicine formulations have also shown efficacy in alleviating symptoms. (**correct**)	181 (34.81)
K7. For patients with symptomatic adenomyosis seeking definitive resolution, hysterectomy may be considered. (**correct**)	147 (28.27)
K8. Adenomyosis can potentially contribute to infertility. (**correct**)	188 (36.15)
K9. Adenomyosis is not typically associated with the induction of psychological or physiological discomfort in women. (**incorrect**)	254 (48.85)
K10. Adenomyosis is a chronic condition necessitating long-term management strategies. (**correct**)	227 (43.65)
K11. A healthy diet, regular exercise, and consistent follow-up can ameliorate the condition of patients with adenomyosis. (**correct**)	241 (46.35)
K12. Adenomyosis does not possess malignant transformation potential. (**incorrect**)	141 (27.12)

Reproductive-age women in this study exhibited varied attitudes toward uterine adenomyosis, with 91.54% concurring on the necessity for fundamental knowledge concerning the disease (A1). 90.77% agreed that attention should be paid to the prevention and treatment of the disease (A2). 92.69% supported the concept that a good lifestyle can help in prevention or treatment (A3) and 88.46% agreed weight control and reduction of abortions can prevent the development of the diseases (A4). When it comes to following medical advice for follow-up and medication, 92.50% had a positive attitude (A5). It is noteworthy that 67.31% considered total hysterectomy is unacceptable (A6), and almost half of them (48.65%) were not sure whether they should opt for conservative surgery or interventional treatment and preferred to follow their doctor’s advice (A6.1; Table 3).

**Table 3:** Attitudes.

	Strongly agree *N* (%)	Agree *N* (%)	Neutral *N* (%)	Disagree *N* (%)	Strongly disagree *N* (%)
A1. You agree that women of childbearing age should have a basic understanding of uterine adenomyosis.	173 (33.27)	303 (58.27)	36 (6.92)	2 (0.38)	6 (1.15)
A2. You believe that uterine adenomyosis can potentially lead to serious adverse outcomes and should be taken seriously in terms of prevention and management, regardless of the diagnosis.	156 (30.00)	316 (60.77)	38 (7.31)	2 (0.38)	8 (1.54)
A3. You acknowledge that adopting a healthy lifestyle, including maintaining good sleep patterns, regular exercise, and a positive mindset, can contribute to the prevention and treatment of uterine adenomyosis.	162 (31.15)	320 (61.54)	32 (6.15)	0	6 (1.15)
A4. You agree that controlling body weight and reducing the number of abortions can help prevent the occurrence and development of uterine adenomyosis.	140 (26.92)	320 (61.54)	48 (9.23)	2 (0.38)	10 (1.92)
A5. It is crucial for the prevention and management of uterine adenomyosis to follow the doctor’s treatment plan, adhere to medication schedules, and attend regular check-ups as advised.	177 (34.04)	304 (58.46)	32 (6.15)	0	7 (1.35)
	**Yes *N* (%)**		**No *N* (%)**		
A6. You think that a total hysterectomy is an acceptable option for curing uterine adenomyosis. (If you choose “no,” please proceed to question 6.1.)	170 (32.69)		350 (67.31)		
	**Conservative surgery (preserving the uterus) *N* (%)**		**Interventional treatment (uterine artery embolization, ablation, etc.) *N* (%)**		**Not sure about the choice, prefer to follow the doctor’s recommendation *N* (%)**
A6.1 If you choose “no,” in cases where medication treatment is ineffective, you prefer what alternative treatment?	82 (15.77)		15 (2.88)		253 (48.65)

34.80% of the participants indicated that they either did not or absolutely not learn about uterine adenomyosis through various sources (P1), and also 38.08% were negative about the therapeutic methods or medication indicated for the disease (P2). On the other hand, 43.46% reported that they would share their experience in the prevention and management of the disease with other women of reproductive age (P3). Positively, more than 75% of the participants were able to detect their abnormal symptoms (P4), seek help from their doctors at the first time (P5), and relieve their bad moods by communicating with their family members, friends, or doctors (P6). In terms of exercise frequency, the largest proportion of respondents only exercised occasionally (42.12%; Table 4).

**Table 4:** Practices.

	Completely compliant *N* (%)	Compliant *N* (%)	Uncertain *N* (%)	Non-compliant *N* (%)	Extremely non-compliant *N* (%)
P1. In your daily life, you seek information and knowledge about the mechanisms, symptoms, and risks of uterine adenomyosis through various sources such as books, the internet, or conversations with doctors.	79 (15.19)	157 (30.19)	103 (19.81)	171 (32.88)	10 (1.92)
P2. You consciously strive to understand the treatment options for uterine adenomyosis, such as learning about different types of surgeries or carefully reading drug labels when purchasing medications.	78 (15.00)	147 (28.27)	97 (18.65)	185 (35.58)	13 (2.50)
P3. You engage in discussions with other women of childbearing age to share insights on preventing and managing uterine adenomyosis.	62 (11.92)	164 (31.54)	133 (25.58)	147 (28.27)	14 (2.69)
P4. You are sensitive to detecting unusual physical symptoms in daily life, such as menstrual pain, prolonged periods, or increased menstrual flow.	79 (15.19)	338 (65.00)	66 (12.69)	28 (5.38)	9 (1.73)
P5. When experiencing unusual symptoms like menstrual pain, prolonged periods, or increased menstrual flow, you promptly seek medical assistance.	81 (15.58)	322 (61.92)	80 (15.38)	28 (5.38)	9 (1.73)
P6. You confide in family and friends or consult with a healthcare professional for psychological support and emotional relief in your daily life.	81 (15.58)	339 (65.19)	59 (11.35)	29 (5.58)	12 (2.31)
	**Always *N* (%)**	**Often *N* (%)**	**Sometimes *N* (%)**	**Occasionally *N* (%)**	**Never *N* (%)**
P7. You maintain a regular exercise routine in your daily life.	54 (10.38)	100 (19.23)	103 (19.81)	219 (42.12)	44 (8.46)

Pearson’s analysis showed that knowledge and the attitudes were positively correlated (r = 0.477, *p* < 0.001), and knowledge and practices were also positively correlated (r = 0.513, *p* < 0.001). Additionally, there was a positive correlation between attitude and practice scores (r = 0.398, *p* < 0.001; Table 5).

**Table 5:** Pearson’s analysis.

	Knowledge	Attitudes	Practices
Knowledge	1		
Attitudes	0.477(*P* < 0.001)	1	
Practices	0.513(*P* < 0.001)	0.398(*P* < 0.001)	1

Multivariate logistic regression demonstrated that high school and vocational school (OR = 3.005, 95% CI: 1.205–7.489, *p* < 0.001), junior college and bachelor’s degree (OR = 6.576, 95% CI: 2.959–14.615, *p* < 0.001), master’s degree and above (OR = 35.996, 95% CI: 13.376–96.866, *p* < 0.001), and diagnosed with uterine adenomyosis (OR = 8.623, 95% CI: 4.634–16.048, *p* < 0.001) were independently associated with sufficient knowledge (Table 6). Knowledge (OR = 1.329, 95% CI: 1.244–1.419, *p* < 0.001), age (OR = 0.952, 95% CI: 0.914–0.991, *p* = 0.016), junior college and bachelor’s degree (OR = 2.387, 95% CI: 1.057–5.391, *p* = 0.036) and master’s degree and above (OR = 3.246, 95% CI: 1.127–9.354, *p* = 0.029) were independently associated with active attitudes (Table 7). Furthermore, knowledge (OR = 1.208, 95% CI: 1.120–1.302, *p* < 0.001), attitudes (OR = 1.218, 95% CI: 1.106–1.341, *p* < 0.001), high school and vocational school (OR = 3.617, 95% CI: 1.654–7.911, *p* = 0.001) and being diagnosed with uterine adenomyosis (OR = 1.942, 95% CI: 1.107–3.409, *p* = 0.021) were independently associated with proactive practice (Table 8).

**Table 6:** Multivariate analysis of knowledge.

	Univariate analysis		Multivariate analysis	
	OR (95% CI)	*P*	OR (95% CI)	*P*
Age (years)	0.954 (0.932 0.977)	<0.001	0.964 (0.925 1.005)	0.087
**BMI (kg/m**^**2**^**)**				
<18.5	ref.			
18.5–23.9	1.139 (0.507 2.559)	0.753		
≥24	0.923 (0.401 2.123)	0.850		
**Residence**				
Rural	ref.			
Urban	1.449 (0.968 2.167)	0.071		
**Marital status**				
Single	ref.		ref.	
Married	0.301 (0.193 0.469)	<0.001	0.715 (0.327 1.560)	0.399
Divorced/Other	0.327 (0.063 1.694)	0.183	0.573 (0.079 4.137)	0.581
**Education level**				
Primary school and below	ref.		ref.	
High school and vocational school	2.344 (1.006 5.460)	0.048	3.005 (1.205 7.489)	0.018
Junior college and bachelor’s degree	4.695 (2.329 9.465)	<0.001	6.576 (2.959 14.615)	<0.001
Master’s degree and above	25.179 (10.862 58.364)	<0.001	35.996 (13.376 96.866)	<0.001
**Average monthly income (CNY)**				
<2,000	ref.			
2,000–5,000	0.667 (0.337 1.318)	0.244		
5,000–10,000	0.878 (0.435 1.773)	0.716		
10,000–20,000	1.615 (0.719 3.624)	0.246		
>20,000	0.886 (0.200 3.916)	0.873		
**Occupation**				
Regular employee/part-time/self-employed	ref.			
Unemployed/retired/other	1.179 (0.798 1.742)	0.408		
**Medical insurance**				
No medical insurance	ref.			
Social insurance only	0.830 (0.378 1.824)	0.642		
Social insurance and other commercial insurance	1.017 (0.451 2.292)	0.968		
Menstrual age (years)	0.980 (0.853 1.126)	0.774		
Menstrual cycle (days)	0.995 (0.978 1.012)	0.559		
Menstrual period (days)	1.103 (0.967 1.259)	0.144		
**Menstrual flow**				
Scanty, spotting	ref.			
Moderate, normal amount	0.968 (0.478 1.959)	0.927		
Heavy, affecting quality of life	1.292 (0.582 2.868)	0.529		
**Count of miscarriage incidents (encompassing both surgical interventions and medication-induced terminations)**				
No history of miscarriage	ref.		ref.	
1 time	0.430 (0.265 0.698)	0.001	0.845 (0.438 1.631)	0.616
2 times	0.353 (0.194 0.642)	0.001	0.944 (0.435 2.049)	0.884
3 times	0.679 (0.311 1.485)	0.333	1.014 (0.363 2.835)	0.978
4 times or more	0.593 (0.183 1.917)	0.383	1.118 (0.271 4.601)	0.878
**Experienced dysmenorrhea symptoms**				
Yes	1.879 (1.270 2.780)	0.002	1.005 (0.607 1.664)	0.985
No	ref.		ref.	
**Diagnosed with uterine adenomyosis**				
Yes	3.112 (2.044 4.737)	<0.001	8.623 (4.634 16.048)	<0.001
No	ref.		ref.	

**Table 7:** Multivariate analysis of attitudes.

	Univariate analysis		Multivariate analysis	
	OR (95% CI)	*P*	OR (95% CI)	*P*
Knowledge	1.359 (1.279 1.443)	<0.001	1.329 (1.244 1.419)	<0.001
Age (years)	0.940 (0.917 0.963)	<0.001	0.952 (0.914 0.991)	0.016
**BMI (kg/m**^**2**^**)**				
<18.5	ref.			
18.5–23.9	0.950 (0.440 2.049)	0.895		
≥24	0.585 (0.263 1.304)	0.190		
**Residence**				
Rural	ref.		ref.	
Urban	2.047 (1.351 3.103)	0.001	1.601 (0.912 2.809)	0.101
**Marital status**				
Single	ref.		ref.	
Married	0.330 (0.212 0.513)	<0.001	1.322 (0.571 3.059)	0.514
Divorced/Other	0.340 (0.066 1.759)	0.198	2.126 (0.292 15.449)	0.456
**Education level**				
Primary school and below	ref.		ref	
High school and vocational school	1.360 (0.579 3.194)	0.480	0.854 (0.327 2.228)	0.747
Junior college and bachelor’s degree	4.585 (2.390 8.794)	<0.001	2.387 (1.057 5.391)	0.036
Master’s degree and above	14.521 (6.643 31.740)	<0.001	3.246 (1.127 9.354)	0.029
**Average monthly income (CNY)**				
<2,000	ref.		ref.	
2,000–5,000	0.438 (0.227 0.845)	0.014	0.655 (0.286 1.502)	0.318
5,000–10,000	0.551 (0.279 1.089)	0.086	0.545 (0.228 1.305)	0.173
10,000–20,000	1.192 (0.544 2.613)	0.661	0.823 (0.297 2.275)	0.707
>20,000	2.132 (0.528 8.598)	0.287	3.463 (0.606 19.777)	0.162
**Occupation**				
Regular employee/part-time/self-employed	ref.			
Unemployed/retired/other	0.887 (0.598 1.316)	0.553		
**Medical insurance**				
No medical insurance	ref.			
Social insurance only	0.803 (0.365 1.767)	0.586		
Social insurance and other commercial insurance	1.158 (0.515 2.603)	0.723		
Menstrual age (years)	0.907 (0.788 1.044)	0.174		
Menstrual cycle (days)	1.012 (0.993 1.030)	0.213		
Menstrual period (days)	1.040 (0.912 1.185)	0.559		
**Menstrual flow**				
Scanty, spotting	ref.			
Moderate, normal amount	1.083 (0.536 2.190)	0.824		
Heavy, affecting quality of life	0.947 (0.420 2.134)	0.895		
**Count of miscarriage incidents (encompassing both surgical interventions and medication-induced terminations)**				
No history of miscarriage	ref.		ref.	
1 time	0.519 (0.326 0.826)	0.006	1.106 (0.578 2.118)	0.760
2 times	0.346 (0.190 0.631)	0.001	0.881 (0.403 1.926)	0.750
3 times	0.343 (0.137 0.861)	0.023	0.666 (0.223 1.993)	0.467
4 times or more	0.801 (0.266 2.417)	0.694	1.372 (0.325 5.801)	0.667
**Experienced dysmenorrhea symptoms**				
Yes	0.992 (0.680 1.447)	0.966		
No	ref.			
**Diagnosed with uterine adenomyosis**				
Yes	1.020 (0.658 1.579)	0.931		
No	ref.			

**Table 8:** Multivariate analysis of practices.

	Univariate analysis		Multivariate analysis	
	OR (95% CI)	*P*	OR (95% CI)	*P*
Knowledge	1.336 (1.259 1.418)	<0.001	1.208 (1.120 1.302)	<0.001
Attitudes	1.343 (1.245 1.449)	<0.001	1.218 (1.106 1.341)	<0.001
Age (years)	1.001 (0.979 1.024)	0.922		
**BMI (kg/m**^**2**^**)**				
<18.5	ref.			
18.5–23.9	0.797 (0.368 1.724)	0.564		
≥24	0.726 (0.328 1.606)	0.429		
**Residence**				
Rural	ref.			
Urban	1.449 (0.968 2.167)	0.071		
**Marital status**				
Single	ref.			
Married	0.763 (0.484 1.204)	0.246		
Divorced/Other	0.657 (0.126 3.422)	0.618		
**Education level**				
Primary school and below	ref.		ref.	
High school and vocational school	3.391 (1.706 6.739)	<0.001	3.617 (1.654 7.911)	0.001
Junior college and bachelor’s degree	2.518 (1.391 4.560)	0.002	1.640 (0.810 3.324)	0.169
Master’s degree and above	5.250 (2.558 40.776)	<0.001	1.805 (0.721 4.518)	0.207
**Average monthly income (CNY)**				
<2,000	ref.			
2,000–5,000	0.821 (0.412 1.635)	0.574		
5,000–10,000	1.000 (0.491 2.038)	1.000		
10,000–20,000	1.333 (0.583 3.049)	0.495		
>20,000	0.571 (0.107 3.041)	0.512		
**Occupation**				
Regular employee/part-time/self-employed	ref.			
Unemployed/retired/other	0.819 (0.550 1.221)	0.327		
**Medical insurance**				
No medical insurance	ref.			
Social insurance only	1.132 (0.490 2.615)	0.772		
Social insurance and other commercial insurance	1.462 (0.618 3.456)	0.387		
Menstrual age (years)	1.035 (0.902 1.189)	0.622		
Menstrual cycle (days)	0.984 (0.954 1.014)	0.283		
Menstrual period (days)	1.165 (1.020 n1.331)	0.024	1.128 (0.964 1.320)	0.134
**Menstrual flow**				
Scanty, spotting	ref.			
Moderate, normal amount	0.706 (0.358 1.395)	0.317		
Heavy, affecting quality of life	1.309 (0.608 2.819)	0.491		
**Count of miscarriage incidents (encompassing both surgical interventions and medication-induced terminations)**				
No history of miscarriage	ref.			
1 time	0.721 (0.451 1.153)	0.172		
2 times	0.573 (0.323 1.018)	0.058		
3 times	1.692 (0.816 3.507)	0.158		
4 times or more	1.071 (0.354 3.242)	0.903		
**Experienced dysmenorrhea symptoms**				
Yes	1.956 (1.321 2.897)	0.001	1.445 (0.887 2.357)	0.140
No	ref.		ref.	
**Diagnosed with uterine adenomyosis**				
Yes	3.112 (2.044 4.737)	<0.001	1.942 (1.107 3.409)	0.021
No	ref.		ref.	

**Figure 1:**
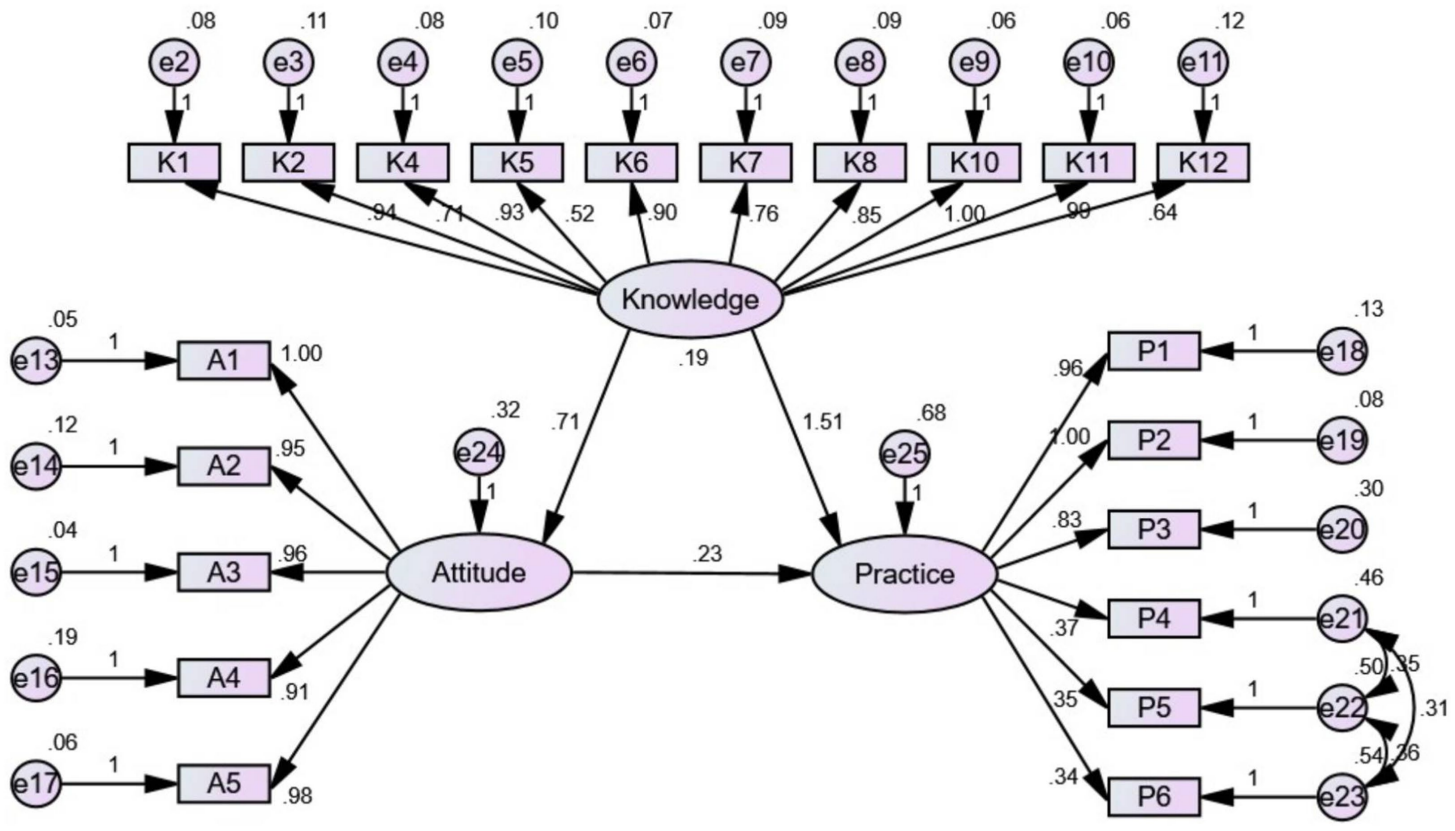
The KAP structural equation model.

The structural equation model demonstrated that knowledge had direct effects on attitudes and practices, as indicated by a path coefficient of 0.714 (*p* < 0.001) and 1.510 (p < 0.001), respectively. Moreover, attitudes had direct effects on practices, with a path coefficient of 0.226 (*p* = 0.001; Table 9; Figure 1). The fitting index of the structural model (CMIN/DF = 2.822; RMSEA = 0.059; IFI = 0.966; TLI = 0.961; CFI = 0.966) outperformed the respective threshold value, signifying that the data satisfactorily fit the structural model (Table 10).

**Table 9:** Test results of the hypothesis.

			Estimate	*P*
Attitude	←	Knowledge	0.714	<0.001
Practice	←	Attitude	0.226	0.001
Practice	←	Knowledge	1.510	<0.001

**Table 10:** Model fitness indices for the KAP structural equation model.

Indicators	Reference	Actual
CMIN/DF	1–3: Excellent, 3–5: Good	2.822
RMSEA	<0.08: Good	0.059
IFI	>0.8: Good	0.966
TLI	>0.8: Good	0.961
CFI	>0.8: Good	0.966

## Discussion

The findings revealed that reproductive-age women have insufficient knowledge, passive attitudes, and poor practices toward the uterine adenomyosis. Comprehensive training programs are needed to improve reproductive-age women practices in this area.

The results of the knowledge dimension reveal several deficiencies in understanding among participants regarding uterine adenomyosis. Notably, a substantial proportion of respondents provided incorrect answers to key aspects of the condition, such as its association with reproductive-age women, its potential impact on fertility, and the chronic nature of the condition requiring long-term management (13). These gaps in knowledge can have significant implications for clinical practice, as misinformation may lead to delayed diagnosis, inappropriate treatment choices, and heightened psychological distress among affected individuals (14). To address these deficiencies, targeted educational interventions are warranted, focusing on disseminating accurate information about uterine adenomyosis, its clinical manifestations, diagnostic approaches, and available management options (6). Previous studies have demonstrated the effectiveness of educational interventions in improving knowledge and awareness among women with reproductive health conditions (15, 16). Therefore, healthcare providers should consider implementing evidence-based educational programs to enhance the understanding of uterine adenomyosis among both healthcare professionals and the general population (17, 18).

The findings from the attitudes dimension highlight both positive and concerning aspects of participants’ attitudes toward uterine adenomyosis. While a majority of respondents express agreement on the importance of women of childbearing age having a basic understanding of the condition and recognizing the need for prevention and management, there are notable gaps in understanding regarding specific aspects (19). For instance, a substantial number of participants disagree or strongly disagree that a total hysterectomy is an acceptable option for curing uterine adenomyosis (20). This discrepancy suggests a potential lack of awareness or reluctance to consider certain treatment options among respondents (6). To address this, healthcare professionals should prioritize patient education level on the range of available treatments, emphasizing the benefits and risks associated with each (21). It is crucial to involve patients in shared decision-making processes, ensuring they have a comprehensive understanding of treatment options and are actively engaged in choosing the most suitable approach for their individual circumstances (22). This aligns with the patient-centered care approach advocated in the literatures, which emphasizes the importance of involving patients in decision-making processes to enhance treatment adherence and overall satisfaction (23–25).

The results of practice dimension provided insights into the daily behaviors and actions of participants regarding uterine adenomyosis. While a significant number of respondents exhibit compliance in seeking information, understanding treatment options, and being sensitive to unusual symptoms, there are notable deficiencies in certain areas, particularly in maintaining a regular exercise routine. Regular exercise has been consistently associated with improved reproductive health outcomes, including a potential reduction in the risk of adenomyosis (26, 27). The findings suggest a need for targeted interventions to promote and facilitate regular exercise among women of childbearing age. Healthcare professionals should emphasize the role of physical activity in preventing and managing uterine adenomyosis, incorporating personalized exercise plans into routine care discussions. Moreover, collaborative efforts with fitness professionals and community-based initiatives may enhance the accessibility and sustainability of exercise programs. This aligns with the broader literature emphasizing the importance of lifestyle interventions in reproductive health, particularly for conditions influenced by modifiable risk factors (28, 29).

The multivariate logistic regression findings highlight the independent effects of educational qualifications and the diagnosis of uterine adenomyosis on knowledge. These factors should be considered in the development of educational materials and strategies. Additionally, the association between knowledge, attitude, and practice scores with age and education level emphasizes the need for age-appropriate and education level-specific interventions.

Furthermore, the correlation between knowledge, attitude, and practice scores suggests a comprehensive approach to intervention. Enhancing knowledge not only positively influences attitudes but also contributes to more proactive health practices. The positive correlation aligns with the health belief model, which posits that individuals with greater knowledge are more likely to engage in preventive health behaviors (30–32). The structural model analysis provides further insights into the interplay between knowledge, attitude, and practice. The direct positive effects of knowledge on attitude and practice, as well as the influence of attitude on practice, emphasize the importance of addressing knowledge gaps to improve attitudes and, consequently, health practices. Interventions should focus not only on increasing knowledge but also on fostering positive attitudes toward uterine adenomyosis, as attitudes play a crucial role in shaping health behaviors (33, 34).

One limitation of this study is its reliance on a web-based cross-sectional design, which may introduce selection bias as it primarily captures responses from individuals with internet access. Additionally, the use of self-reported data may introduce recall and response biases, as participants might not accurately recall or report their knowledge, attitudes, and practices regarding uterine adenomyosis. Despite these limitations, the study’s strengths include its large sample size, statistical rigor, and use of a SEM, providing valuable insights into the associations between knowledge, attitudes, and practices related to uterine adenomyosis. In light of the study’s outcomes, strategic interventions are warranted to enhance clinical practices related to uterine adenomyosis among reproductive-age women.

## Data availability statement

The original contributions presented in the study are included in the article/supplementary material, further inquiries can be directed to the corresponding author.

## Ethics statement

The studies involving humans were approved by the Ethics Committee of the Second Hospital of Hebei Medical University (Approval No. 2023-R010). The studies were conducted in accordance with the local legislation and institutional requirements. The participants provided their written informed consent to participate in this study.

## Author contributions

RR: Data curation, Writing – review & editing. HL: Data curation, Writing – review & editing. JZ: Data curation, Writing – review & editing. XL: Data curation, Writing – review & editing. LY: Conceptualization, Formal analysis, Writing – review & editing. DL: Conceptualization, Formal analysis, Writing – review & editing. SS: Conceptualization, Formal Analysis, Writing – review & editing. BS: Data curation, Formal analysis, Writing – original draft. JJ: Data curation, Formal analysis, Writing – original draft.

## Footnotes

^1^ http://www.sojump.com
